# On the sighted ancestry of blindness – exceptionally preserved eyes of Mesozoic polychelidan lobsters

**DOI:** 10.1186/s40851-016-0049-0

**Published:** 2016-07-16

**Authors:** Denis Audo, Joachim T. Haug, Carolin Haug, Sylvain Charbonnier, Günter Schweigert, Carsten H. G. Müller, Steffen Harzsch

**Affiliations:** Université de Rennes 1, EA 7316, 263 Avenue du Général Leclerc CS 74205, 35042 Rennes Cedex, France; Centre de Recherche sur la Paléobiodiversité et les Paléoenvironnements (CR2P, UMR 7207), Muséum national d’Histoire naturelle, Sorbonne Universités, UPMC, CNRS, 8 rue Buffon, F-75005 Paris, France; Department Biologie II und GeoBio-Center, Ludwig-Maximilians-Universität München, Biozentrum der LMU, Großhaderner Str. 2, 82152 Planegg-Martinsried, Germany; Staatliches Museum für Naturkunde Stuttgart, Rosenstein 1, 70191 Stuttgart, Germany; Zoologisches Institut und Museum, Lehrstuhl Allgemeine und Systematische Zoologie, Ernst-Moritz-Arndt-Universität Greifswald, Anklamer Str. 20, 17487 Greifswald, Germany; Zoologisches Institut und Museum, Lehrstuhl Cytologie und Evolutionsbiologie, Ernst-Moritz-Arndt-Universität Greifswald, Soldmannstr. 23, 17489 Greifswald, Germany

**Keywords:** Polychelida, Solnhofen, La Voulte-sur-Rhône, Osteno, Nusplingen, Posidonia Shale, Heterochrony, Deep-sea adaptations, Superposition eyes

## Abstract

**Background:**

Modern representatives of Polychelida (Polychelidae) are considered to be entirely blind and have largely reduced eyes, possibly as an adaptation to deep-sea environments. Fossil species of Polychelida, however, appear to have well-developed compound eyes preserved as anterior bulges with distinct sculpturation.

**Methods:**

We documented the shapes and sizes of eyes and ommatidia based upon exceptionally preserved fossil polychelidans from Binton (Hettangian, United-Kingdom), Osteno (Sinemurian, Italy), Posidonia Shale (Toarcian, Germany), La Voulte-sur-Rhône (Callovian, France), and Solnhofen-type plattenkalks (Kimmeridgian-Tithonian, Germany). For purposes of comparison, sizes of the eyes of several other polychelidans without preserved ommatidia were documented. Sizes of ommatidia and eyes were statistically compared against carapace length, taxonomic group, and outcrop.

**Results:**

Nine species possess eyes with square facets; *Rosenfeldia oppeli* (Woodward, 1866), however, displays hexagonal facets. The sizes of eyes and ommatidia are a function of carapace length. No significant differences were discerned between polychelidans from different outcrops; Eryonidae, however, have significantly smaller eyes than other groups.

**Discussion:**

Fossil eyes bearing square facets are similar to the reflective superposition eyes found in many extant decapods. As such, they are the earliest example of superposition eyes. As reflective superposition is considered plesiomorphic for Reptantia, this optic type was probably retained in Polychelida. The two smallest specimens, a *Palaeopentacheles roettenbacheri* (Münster, 1839) and a *Hellerocaris falloti* (Van Straelen, 1923), are interpreted as juveniles. Both possess square-shaped facets, a typical post-larval feature. The eye morphology of these small specimens, which are far smaller than many extant eryoneicus larvae, suggests that Jurassic polychelidans did not develop via giant eryoneicus larvae. In contrast, another species we examined, *Rosenfeldia oppeli* (Woodward, 1866), did not possess square-shaped facets, but rather hexagonal ones, which suggests that this species did not possess reflective superposition eyes. The hexagonal facets may indicate either another type of superposition eye (refractive or parabolic superposition), or an apposition eye. As decapod larvae possess apposition eyes with hexagonal facets, it is most parsimonious to consider eyes of *R. oppeli* as apposition eyes evolved through paedomorphic heterochrony.

**Conclusion:**

Polychelidan probably originally had reflective superposition. *R. oppeli*, however, probably gained apposition eyes through paedomorphosis.

## Background

### Eyes in the fossil record

The study of eye structures in fossil arthropods has long been limited to that of highly calcified trilobites [1]. Due to their sturdy outer surface, eyes and interior structures, such as substructures of the lenses, are regularly preserved in trilobite fossils [2]. The investigation of exceptionally well-preserved eye structures in less heavily calcified arthropod taxa (e.g., crustaceans) has attracted a great deal of scientific attention in recent years. Although the preservation of such an apparently fragile organ would appear to be exceptional, the preservation of eye structures in sclerotized arthropods is not uncommon. The good preservation of some such fossil eyes has, in certain cases, even allowed the partial reconstruction of putative optical properties [3–6]. Such studies focused on functional aspects of eyes possessed by early representatives of different arthropod lineages to infer possible ancestral features, and thus to shed light on the early evolution of eye structures in sclerotized arthropods. These studies included eyes of presumably early chelicerates [7–11] which, however, have been alternatively interpreted as representatives of the lineage towards Euarthropoda [12, 13] (see also Haug et al. critical review [9]).

These early arthropods include a number of spectacular taxa, such as (1) *Anomalocaris* sp. from Emu Bay Shale [4] which is one of the arthropods with the highest known number of ommatidia in the compound eye, or (2) the early chelicerate *Leanchoilia superlata* Walcott, 1912 which initially was believed to have possessed two pairs of compound eyes [14], but later was suggested to have carried “just” one pair of bilobed compound eyes [10] (as do other species of *Leanchoilia* [15]). In addition, favorable incidents during fossilization of Early Cambrian biota not only led to the preservation of prominent epidermal sense organs (e.g., compound eyes, cuticular sensilla), but also yielded information on the anatomy, as was demonstrated most recently in the early sclerotized arthropod from Chengjiang *Fuxianhuia protensa* Hou, 1987, which displayed a tripartite brain and optic neuropil [16]. Further studies also included early representatives of the mandibulate lineage other than trilobites; i.e., softer (non-calcified) and smaller fossils of early representatives Crustacea *sensu lato* [17] discovered as uncompressed fossils. In these, the cuticle became impregnated by calcium phosphate, in a so-called Orsten-type preservation [18]. Among fossilized arthropods, trilobites and Orsten crustaceans share the advantage of being preserved extraordinarily well, retaining most of their original volume [18].

Besides these early derivatives of different evolutionary lineages, fossilized eye structures of representatives of extant groups have been described in Orsten-type preservation, for example the compound eyes of an ostracod [19], an achelate phyllosoma larva [3] and a possible maxillopod [17]. Eyes preserved uncompressed in three dimensions, i.e. in their original volume, are also reported in many arthropods from the La Voulte-sur-Rhône nodules, notably in thylacocephalans [20–24] and in glypheid lobsters [25].

A further source of exquisitely preserved fossils comes from the plattenkalks. Although the body volume of these fossils was not preserved entirely, as for instance in Orsten’s or La Voulte’s fossils, at least some specimens may show aspects of their original volume [26]), or even appear almost uncompressed [27]. Specimens with preserved eyes have been described from the famous Solnhofen-type plattenkalks of southern Germany (e.g. an isopod). Additional, although not very well-preserved examples of fossil eyes include those of benthesicymid shrimps [28] and of cirolanid isopods [29, 30] from the Late Cretaceous Sahel Alma Lagerstätte (Lebanon).

### Eyes in polychelidan lobsters

Polychelidan lobsters are ascribed to Reptantia, a group comprising mostly benthic crustaceans with elongated pleon, such as spiny, slipper or squat lobsters, crayfishes, hermit crabs, and true crabs. Among Reptantia, Polychelida is probably the sister group to Eureptantia comprising all remaining groups of Reptantia [31]. The predominantly benthic polychelidans have retained some plesiomorphic characters from the ground pattern of natantian shrimps, such as the triangular telson. Polychelida is thus a key group for reconstructing character evolution in Reptantia, and Decapoda as a whole.

Adults of extant species of Polychelida are all thought to be blind and to inhabit deep-sea ecosystems [32]. Their larvae, however, may retain functional eyes [33] which may degenerate over the course of development. In adults, the eye stalks are still present (except in *Willemoesia* Grote, 1873), but the corneae are always reduced; despite this, live specimens of *Polycheles typhlops* Heller, 1862 still reacted to the intensity of light [34]. However, it is known that in the fossil record, many specimens of polychelidan lobsters possessing eyes are present. Even the oldest occurrence of fossil polychelidan, *Coleia uzume* [35] from Japan and *Tetrachela raiblana* [36] from Italy and Austria, both Carnian (Late Triassic), seem to have had developed eyes as, although the eyes themselves may not have been preserved, their carapace margins are incised by developed ocular incisions [35, 36]. Despite being documented fossilized eyes have not been sufficiently discussed in an evolutionary context [37–40]. Spence Bate [41] was the first to stress the occurrence of eyes in some fossil forms, and assumed their reduction in extant species. More recently, Schweigert & Dietl [42] illustrated an eye-bearing specimen of *Palaeopolycheles longipes* (Fraas, 1855) from the Nusplingen Plattenkalk and discussed its ommatidia. On the basis of the ommatidial facets of *Paleopopolycheles longipes* [24] as reported by Schweigert & Dietl [42], and its phylogenetic position, Ahyong [43] suggested that coleiids may have already shifted to deep water, in which case the stem polychelids may have also evolved in deep habitats.

#### Palaeoenvironments and associated faunas

##### Hettangian of Binton (United-Kingdom)

The outcrop of Binton (Warwickshire, United-Kingdom) corresponds to the Wilmcote Limestone member of the basal Blue Lias Formation dating from Rhaetian to Hettangian. The Blue Lias Formation was deposited in a shallow epicontinental sea covering England, which was “normally no more than a few tens of meters deep” [44]. The Blue Lias Formation yielded other fossils, including ichthyosaurs.

##### Sinemurian of Osteno (Italy)

The main outcrop is a quarry along the lake Lugano (Ceresio), near Osteno village (Como, Italy). It is a Fossil-Lagerstätte (outcrop with exceptional preservation of remains in connection, with frequent soft-part preservation). It has yielded some terrestrial plants (allochthonous). It more importantly yielded an important exceptionally preserved marine fauna dominated by crustaceans and thylacocephalans, which also included fishes, cephalopods, polychaetes, and acorn worms [45, 46]. The palaeoenvironment is not well-constrained; however, Teruzzi [47] pointed out similarities in the sponge community with those developing at the limit between the modern neritic–pelagic basins. Osteno was therefore probably deposited in a relatively deep palaeoenvironment.

##### Toarcian of Holzmaden and Gomaringen (Germany)

The outcrops of Holzmaden and Gomaringen (Baden-Württemberg, Germany) are parts of the Posidonia Shale Formation. This formation represents a Fossil-Lagerstätte celebrated for its exquisite preservation of an abundant fauna of marine reptiles that included very large specimens of ichthyosaurs, plesiosaurs, and crocodiles. The Posidonia Shale Formation also yielded some terrestrial tetrapods, numerous bony fishes, sharks, crustaceans, crinoids attached to driftwood, ammonites, brachiopods, numerous bivalves and coleoids, including some with soft parts preserved. Some plant fragments (in addition to driftwood) also occur [48]. Deposition of the Posidonia Shale and the nature of its palaeoenvironment are subject of debate. Bottom water and/or sediment seem to have been often anoxic, with short period of oxygenation. The Posidonia Shale was possibly deposited at a depth of around 50–150 m [49]. The anoxia was possibly a result of significant productivity in the overlying water, which may have caused the benthic habitat to be quite dark.

##### Bajocian-Bathonian of Monte Fallano (Italy)

Monte Fallano is a recently discovered Fossil-Lagerstätte. Its flat-bedded, micritic limestones are similar to those of the plattenkalks of southern Germany; it was therefore possibly deposited in a lagoon fringed by reefs in which the (parautochthonous) fauna lived, similarly to the younger southern German plattenkalks. This outcrop yielded terrestrial plants, insects, numerous fishes, including coral-feeding pycnodonts and crustaceans [50].

##### Callovian of La Voulte-sur-Rhône (France)

The Fossil-Lagerstätte of La Voulte-sur-Rhône is celebrated for its rich fauna preserving cephalopods with their soft parts, abundant ophiuroids, numerous thylacocephalans (with huge eyes), numerous crustaceans, sea spiders, coelacanths and sharks. The La Voulte Lagerstätte is thought to correspond to a deep-water environment, based upon geological and palaeontological evidence (absence of orientation of epizoans on sponges in adjacent outcrops, presence of deep-sea cephalopods) summarized in Charbonnier [22, 51]. Polychelidans were probably autochthonous to this outcrop [24].

##### Kimmeridgian-Tithonian plattenkalks of southern Germany and Cerin (France)

The southern Germany plattenkalks are among the most famous Fossil-Lagerstätten. They are often referred as “Solnhofen-type” outcrops. They were deposited in the regressive trend of the end of Jurassic in small lagoons. Cerin belonged to the same palaeogeographic region [52]. These plattenkalks preserved some terrestrial taxa with plants, insects and tetrapods (e.g. *Archaeopteryx*) and a rich marine fauna dominated by fishes and arthropods, and local echinoderms, also including jellyfishes, brachiopods, annelids, and acorn worms [53]. Most of the fauna was probably parautochthonous to allochthonous, since traces of life such as bioturbation, are rare in these plattenkalks. The plattenkalks were probably formed in a quite shallow palaeoenvironment, ranging from 20 to 60 m in depth [54].

## Methods

### Objectives

**Fig. 1:**
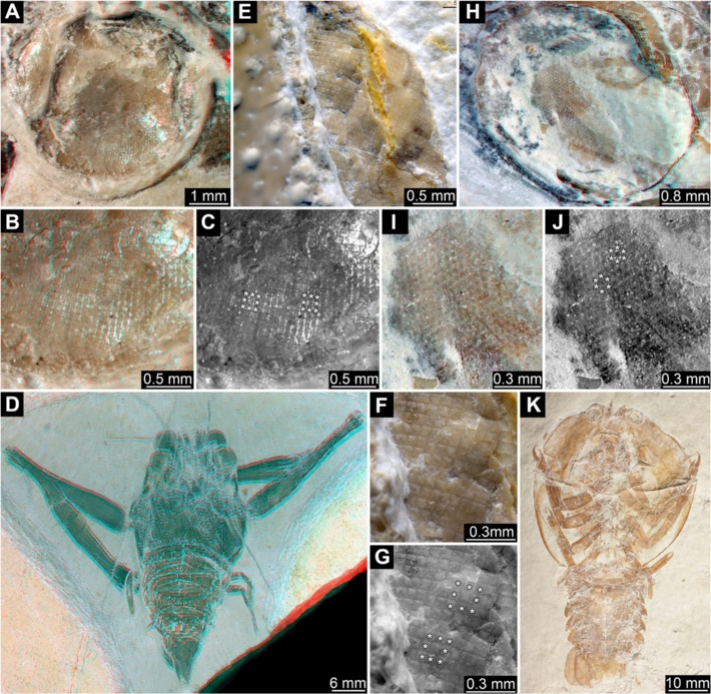
Late Jurassic Polychelida with preserved compound eye structures. **a**-**d** *Palaeopolycheles longipes*, SMNS 63724. **e**-**g**. *Knebelia totoroi*, SMNS 67916. **h**-**k**. *Rosenfeldia oppeli*, SMNS 66004. **a**. Presumed compound eye with partially preserved cornea. **b**. Detail of the cornea with square facets. **c**. Same detail as in B; note regular alignment of square facets, see asterisks (*) indicating eight facets surrounding a central one. **d**. Overview image of entire specimen. **e**-**g**. Details of square corneal facets, note asterisk (*) marking some facets in G. **h**. Presumed compound eye with in part preserved cornea. **i**-**j**. Detail of corneal facets with apparently hexagonal profile, note asterisks (*) marking some facets in J, each facet is surrounded by six neighbored ones. **k**. Overview of entire specimen. **a**-**b**, **d**, **h**-**i** are red-cyan stereo images. Please use red-cyan stereo glasses to view

**Fig. 2:**
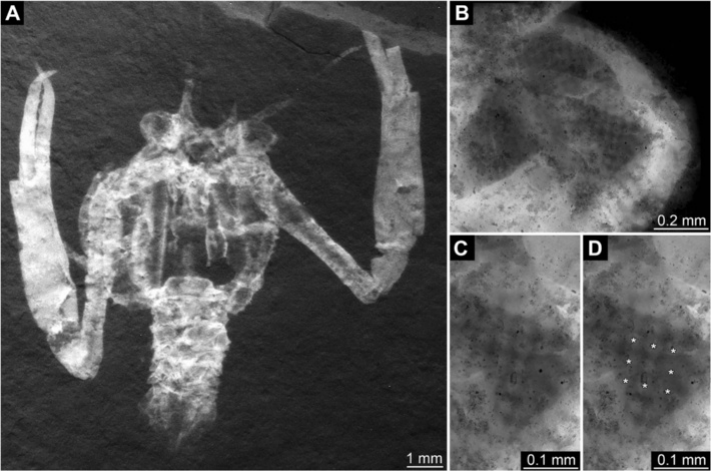
Late Jurassic Polychelida with preserved compound eyes, continued. **a**. Specimen of *Palaeonpentacheles roettenbacheri* (SMNS 67903). **b**. Close-up on right eye. **c**. Close-up on facetted region. **d**. Same details as C, note asterisks (*) revealing each square facet being surrounded by eight neighbored facets. All images are fluorescence composite images

**Fig. 3:**
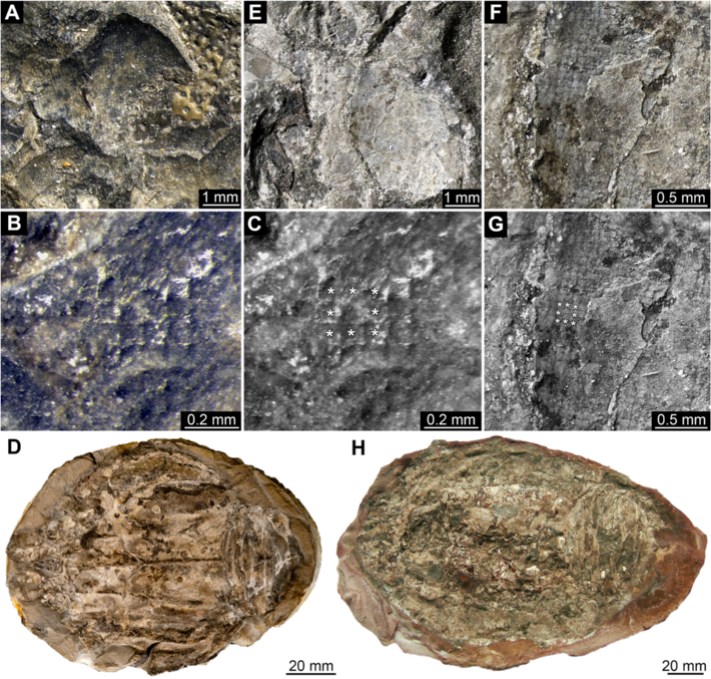
Middle Jurassic (Callovian) Polychelida (*Proeryon giganteus*) from La Voulte-sur-Rhône (France) displaying preserved eye structures. **a**-**d**. Specimen UJF-ID.11547. **e**-**h**. Specimen UPMC-248. **a**, **e**. Presumed eye structures. **b**-**c**, **f**-**g**. Details of square facets, note that each square facet is surrounded by eight adjacent facets, see asterisks (*) in **c** (same image as **b**) and **g** (same image as **e**). **d**, **h**. Overview of entire specimens

**Fig. 4:**
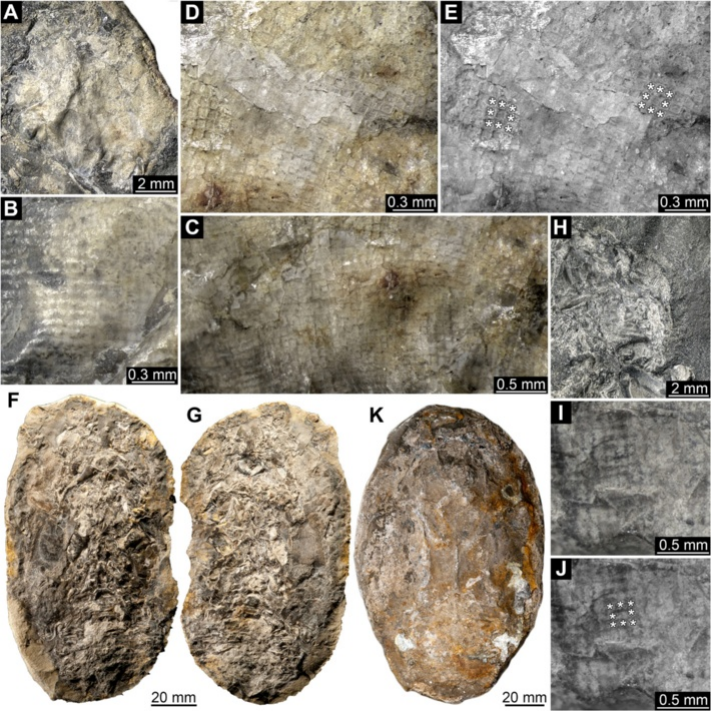
Middle Jurassic (Callovian) Polychelida (*Proeryon giganteus*) from La Voulte-sur-Rhône (France) with preserved eye structures. **a**-**g**. Specimen UJF-ID.14020. **h**-**k**. Specimen FSL 170603. **a**, **h**. Presumed remains of compound eyes. **b**-**e**, **i**-**j**. Details of square facets; note that each square facet is surrounded by eight neighbor facets, see asterisks (*) in **e** (same image as **d**) and **j** (same image as **i**). **f**, **g**. Overview of part and counterpart. **k**. Overview of entire specimen

**Fig. 5:**
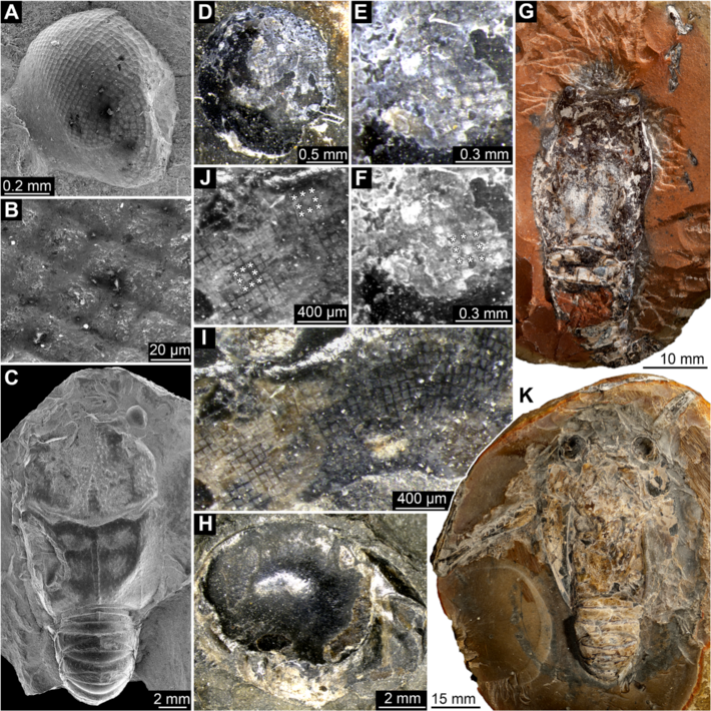
Middle Jurassic (Callovian) Polychelida from La Voulte-sur-Rhône (France) with preserved compound eye structures, continued: *Hellerocaris falloti* **a**-**g** and *Willemoesicaris ovalis* **h**-**k. a**-**c**. Specimen MNHN.F.A50709, SEM images (Philippe Loubry). **d**-**g**. Specimen FSL 710108. **h**-**k**. Specimen MNHN.F.A29521. **a**, **d**, **h**. Overview of compound eye structure. **b**, **e**-**f**, **i**-**j**. Details of square facets, note that each square facet is surrounded by eight neighbor facets, see asterisks (*) in **f** (same image as **e**) and **j** (part of **i**).**c**, **g**, **k**. Overview of entire specimens

**Fig. 6:**
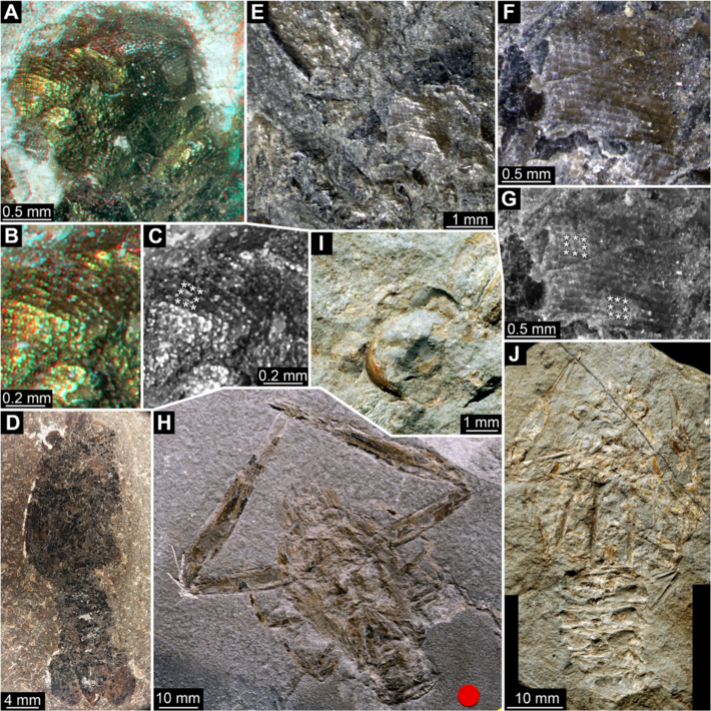
Early Jurassic Polychelida with preserved compound eye structures. **a**-**d**. *Gabaleryon* sp. specimen SMNS 67631 from the Toarcian of Gomaringen. **e**-**h**. *Coleia viallii*, holotype MSNM i3368 from the Sinemurian of Osteno. **i**-**j**. *Coleia barrovensis*, specimen NHMUK.I6589 (photo © NHMUK) from the Hettangian of Binton. **a**, **e**, **i**. Overview of presumed compound eye structures. **b**-**c**, **f**-**g**. Details of square facets, note that each square facet is surrounded by eight neighbor facets, see asterisks (*) in **c** (same image as **b**) and **g** (same image as **f**). **d**, **h**, **j**. Overview of entire specimens

**Fig. 7:**
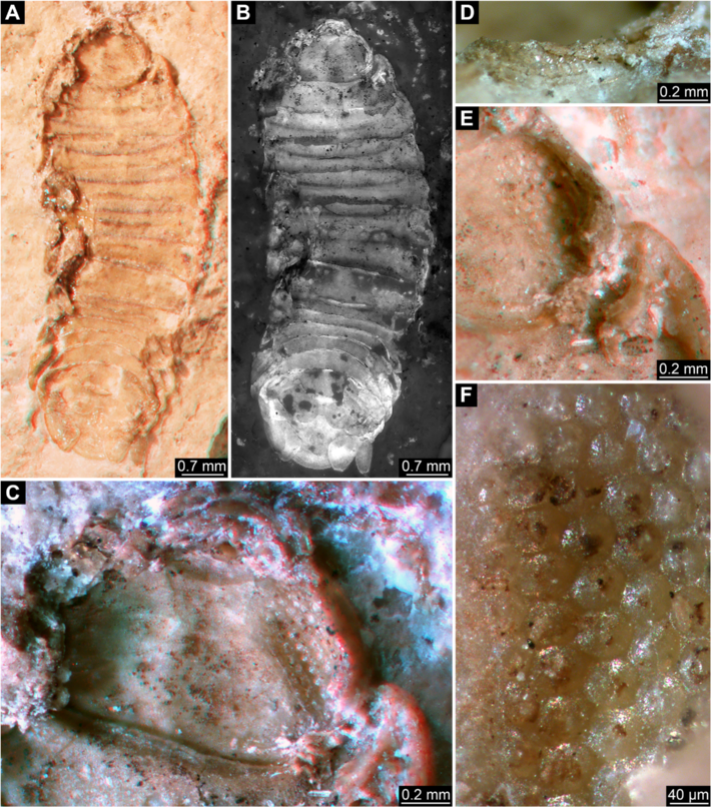
Comparison with eye-bearing arthropods other than Polychelida: 1. Isopoda. **a**, **b**. Overview of specimen of *Palaega nusplingensis* from the Nusplingen Plattenkalk, SMNS 65512. Note that the specimen is mainly seen from the inside. **c**. Close-up of head. **d**. Close-up on a small part of the compound eye seen on the outside. **e**. Close-up on left side of the head, from a more oblique angle than in **c. f**. Close-up of hexagonal facets. **a**, **c** and **e** are red-cyan stereo-images (use red-cyan stereo glasses)

**Fig. 8:**
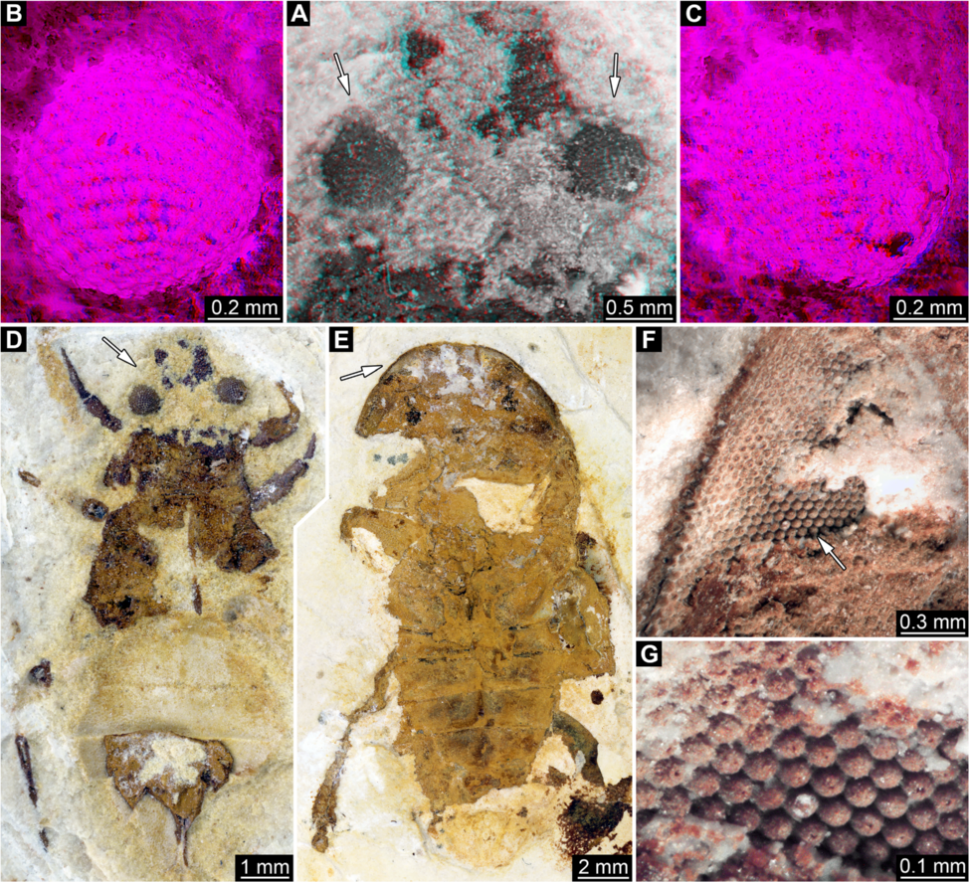
Comparison with eye-bearing arthropods other than Polychelida: 2. Hexapoda, Pterygota from the Santana Formation, Brazil. **a**-**d**. Specimen of an undetermined heteropteran species with hexagonal facets. **e**-**g**. Specimen of an undetermined notonectid species with hexagonal facets. (**a**, **b**, **c**: use red-cyan stereo glasses)

We present new data on eye structures of fossil Polychelida. This includes re-description of the specimen of *Palaeopolycheles longipes* previously shown in Schweigert & Dietl [42]. We also illustrate new specimens of Mesozoic Polychelida from Solnhofen-type plattenkalks (Late Jurassic; Figs. 1 and 2), La Voulte-sur-Rhône (Middle Jurassic: Figs. 3, 4 and 5), and Gomaringen, Osteno and Binton (Early Jurassic: Fig. 6) and compare them to other fossil arthropods, whose living relatives possess well-developed eyes (Figs. 7 and 8). Based on these observations, we discuss the evolution of eye types and optical mechanisms within Polychelida and propose new ideas on the visual ecology of fossil polychelidans.

### Origin of specimens

In total, fourteen specimens of polychelidan lobsters with distinct eye structures and preserved facets were examined (Tables 1 and 2):

**Table 1:** Measurements and estimations of eyes proprieties in studied samples of polychelidan with preserved ommatidia

			*d*: Eye diameter (μm)	*a*: Om-matidia size (μm)	Ommatidia shape	Approximation of eye geometry used	*n*: Longest row of ommatidia	Opening angle of ommatidia (degree)	*se*: Surface of eye (μm²)	*so*: surface of one ommatidium (μm²)	Number of ommatidia
		Formula applied →	Measured		Flat	$\frac{d}{a}$	nul, by definition	$\pi \times {\left(\frac{d}{2}\right)}^2$	Quadrate:	a²	$\frac{se}{so}$
Age	Species ↓	Specimen ↓			Hemispheric	$\frac{\pi \times d}{2\times a}$	$\frac{180}{n}$	$2\pi \times {\left(\frac{d}{2}\right)}^2$	Hexagonal:	$$ \begin{array}{l}3\sqrt{3}\hfill \\ {}2\hfill \end{array}\times {\left(\begin{array}{l}a\hfill \\ {}2\hfill \end{array}\right)}^2 $$	
Late Jurassic	*Rosenfeldia oppeli*	SMNS 66004	3,400	53	Hexagonal	Flat	64	0.00	9,079,203	1,824	4,976
						Hemispheric	101	1.79	18,158,406		9,953
	*Palaeopentacheles roettenbacheri*	SMNS 67903	1,100	35	Quadratic	Flat	31	0.00	950,332	1,225	776
						Hemispheric	49	3.65	1,900,664		1,552
	*Palaeopolycheles longipes*	SMNS 63724	4,200	50	Quadratic	Flat	84	0	13,854,424	2,500	5,542
						Hemispheric	132	1.36	27,708,847		11,084
	*Knebelia totoroi*	SMNS 67916	7,200	67.5	Quadratic	Flat	107	0	40,715,041	4,556	8,936
						Hemispheric	168	1.07	81,430,082		17,872
Middle Jurassic	*Proeryon giganteus*	FSL 170603	9,100	90	Quadratic	Flat	101	0	65,038,822	8,100	8,029
						Hemispheric	159	1.13	130,077,644		16,059
		UJF-ID 11547	11,000	88	Quadratic	Flat	125	0	95,033,178	7,744	12,272
						Hemispheric	196	0.92	190,066,356		24,544
		UJF-ID 14020*	11,600	78.8	Quadratic	Flat	147	0	105,683,177	6,209	17,020
						Hemispheric	231	0.78	211,366,354		34,040
		UPMC-248	NA	77	Quadratic	Flat	NA	NA	NA	5,929	NA
						Hemispheric	NA	NA	NA		NA
	*Hellerocaris falloti*	FSL 710108	1,900	62	Quadratic	Flat	31	0	2,835,287	3,844	738
						Hemispheric	48	3.74	5,670,575		1,475
		MNHN.F.A50709*	850	24.25	Quadratic	Flat	35	0	567,450	588	965
						Hemispheric	55	3.27	1,134,900		1,930
	*Willemoesiocaris ovalis*	MNHN.F.A29521*	7,000	68.83	Quadratic	Flat	102	0	38,484,510	4,738	8,122
						Hemispheric	160	1.13	76,969,020		16,245
Early Jurassic	*Gabaleryon sp*.	SMNS 67631	1,850	55	Quadratic	Flat	34	0	2,688,025	3,025	889
						Hemispheric	53	3.41	5,376,050		1,777
	*Coleia viallii*	MSNM.i3368	5600	82	Quadratic	Flat	68	0	24,630,086	6,724	3,663
						Hemispheric	107	1.68	49,260,173		7,326
	*Coleia barrovensis*	NHMUK.I6589	4100	66	Quadratic	Flat	62	0	13,202,543	4,356	3,031
						Hemispheric	98	1.84	26,405,086		6,062

The size of square ommatidial facets corresponds to the measure of their side length; that of hexagonal ommatidial facets corresponds to the distance between to opposite summits of the hexagon, which is in turn equal to two times the side length. The asterisks after specimens registry numbers indicate that given values are based on multiple ommatidia length measurements, in these cases average values are given. Measured values for these specimens are indicated in Table 2. Distances in μm, surfaces in μm^2^

**Table 2:** Measurements and position of ommatidia in specimens having ommatidia of different sizes

	Ommatidia size						
Specimen	Lower	Position on eye	Intermediate	Position on eye	Higher	Position on eye	Mean value
UJF-ID 14020	68	dorsal	76	anterior	92.4	anterior	78.80
MNHN.F.A29521	61	lateral	70	posterior	75.5	posterior	68.83
MNHN.F.A50709	21.5	anterior	27	lateral, dorsal	NA	NA	24.25

Indication is made of the position of measured ommatidia on the surface of the eye. Measurements in μm

1. a specimen of *Coleia barrovensis* M’Coy, 1849 from the Hettangian of Binton (Warwickshire, United Kingdom) preserving eyes and ommatidia, first observed by Woods [38] and which represents the oldest fossil examined in this study;
2. the holotype of *Coleia viallii* Pinna, 1968 from the Sinemurian (Early Jurassic) of Osteno (northern Italy);
3. a specimen of *Gabaleryon* Audo et al. in press from the Toarcian (Early Jurassic) of Gomaringen (southwestern Germany). This specimen is too poorly preserved to be identified to the species level, however, it is the first in which ommatidia are preserved [55];
4. seven specimens from the Callovian (Middle Jurassic) La Voulte-sur-Rhône Lagerstätte (France), among which four specimens of *Proeryon giganteus* [39], two specimens of *Hellerocaris falloti* [39], and one specimen of *Willemoesiocaris ovalis* [39];
5. four specimens from the Late Jurassic Solnhofen-type plattenkalks including one specimen of *Palaeopolycheles longipes* and the holotype of *Knebelia totoroi* Audo et al., 2014b, in which preservation of ommatidia was previously reported by Audo et al. [56], the latter two from Nusplingen (Late Kimmeridgian), a specimen of *Rosenfeldia oppeli* (Woodward, 1866) from Sappenfeld near Eichstätt (Early Tithonian), and a small specimen of *Palaeopentacheles roettenbacheri* (Münster, 1839) from Eichstätt (Early Tithonian) (hence being the youngest occurrences).

All of the above specimens are flattened to a variable degree, including those from La Voulte which retained most of their original volume. To assess the quality of preservation, we compared our sampling of polychelidan fossils with three specimens belonging to different arthropod taxa, such as the isopod *Palaega nusplingensis* Polz, Schweigert & Maisch, 2006 from the Nusplingen Plattenkalk (Germany) or two insects from the Early Cretaceous Santana Formation (Brazil).

For comparison purposes, we also documented 30 additional specimens on which eyes are preserved, but the outline of each ommatidium is not visible (see Table 3). We additionally documented eyes of one isopod and insects that display preservation modes similar to those of fossil polychelidans.

**Table 3:** Measurements of carapace length, eye diameter and ommatidia surface for all studied specimens of polychelidans

Species	Specimen	CL: Carapace Length (μm)	d: Eye diameter (μm)	d/CL	ommatidia surface	Outcrop	Putative palaeodepth	Family
*Coleia barrovensis*	NHMUK.I6589	28000	4100	0.15	4356	Binton	shallow	Coleiidae
*Gabaleryon sp*.	SMNS 67631	18300	1850	0.10	3025	Gomaringen	intermediate	Coleiidae
*Coleia viallii*	MSNM i3368	37000	5600	0.15	6724	Osteno	deep	Coleiidae
*Cycleryon elongatus*	SNSB-BSPG AS I 939	39700	4700	0.12	NA	Southern Germany plattenkalks	shallow	Eryonidae
*Cycleryon elongatus*	SNSB-BSPG AS VI 43	39800	4100	0.10	NA	Southern Germany plattenkalks	shallow	Eryonidae
*Cycleryon orbiculatus*	SMNS 62574	19600	3150	0.16	NA	Southern Germany plattenkalks	shallow	Eryonidae
*Cycleryon orbiculatus*	SMCU F11408	32500	3900	0.12	NA	Southern Germany plattenkalks	shallow	Eryonidae
*Cycleryon orbiculatus*	SNSB-BSPG AS VII 762	15800	2200	0.14	NA	Southern Germany plattenkalks	shallow	Eryonidae
*Cycleryon propinquus*	SMNS (no reg. Number)	62000	4800	0.08	NA	Southern Germany plattenkalks	shallow	Eryonidae
*Cycleryon propinquus*	MNHN.F.B13436	59000	8300	0.14	NA	Southern Germany plattenkalks	shallow	Eryonidae
*Cycleryon propinquus*	SNSB-BSPG 1922 I 35	85000	8500	0.10	NA	Southern Germany plattenkalks	shallow	Eryonidae
*Cycleryon propinquus*	SNSB-BSPG AS VI 42	57000	3000	0.05	NA	Southern Germany plattenkalks	shallow	Eryonidae
*Cycleryon propinquus*	SNSB-BSPG (no reg. Number)	49000	6500	0.13	NA	Southern Germany plattenkalks	shallow	Eryonidae
*Cycleryon propinquus*	JME-SOS 6827	55500	5200	0.09	NA	Southern Germany plattenkalks	shallow	Eryonidae
*Cycleryon propinquus* (*female*)	SNSB-BSPG AS VI 40	73000	5400	0.07	NA	Southern Germany plattenkalks	shallow	Eryonidae
*Cycleryon romani*	FSL 170522	112000	4000	0.04	NA	La-Voulte-sur-Rhône	deep	Eryonidae
*Hellerocaris falloti*	MNHN.F.A50709	6500	850	0.13	588	La-Voulte-sur-Rhône	deep	aff. Polychelidae
*Hellerocaris falloti*	FSL 710108	24500	1900	0.08	3844	La-Voulte-sur-Rhône	deep	aff. Polychelidae
*Hellerocaris falloti*	FSL 170598	25800	2350	0.09	NA	La-Voulte-sur-Rhône	deep	aff. Polychelidae
*Knebelia bilobata*	JME-SOS 6864	40000	4600	0.12	NA	Southern Germany plattenkalks	shallow	Eryonidae
*Knebelia bilobata*	NHMUK.In 28964	54000	4300	0.08	NA	Southern Germany plattenkalks	shallow	Eryonidae
*Knebelia bilobata*	SMNS 70044	43500	4000	0.09	NA	Southern Germany plattenkalks	shallow	Eryonidae
*Knebelia totoroi*	SMNS 67916	68500	7200	0.11	4556	Southern Germany plattenkalks	shallow	Eryonidae
*Palaeopentacheles roettenbacheri*	SMNS 67903	4200	1100	0.26	1225	Southern Germany plattenkalks	shallow	Palaeopentachelidae
*Palaeopentacheles roettenbacheri*	BSPG AS I 989	30000	4900	0.16	NA	Southern Germany plattenkalks	shallow	Palaeopentachelidae
*Palaeopentacheles roettenbacheri*	BSPG AS I 992	26700	3400	0.13	NA	Southern Germany plattenkalks	shallow	Palaeopentachelidae
*Palaeopentacheles roettenbacheri*	SNSB-BSPG (no reg. Number)	9250	900	0.10	NA	Southern Germany plattenkalks	shallow	Palaeopentachelidae
*Palaeopolycheles longipes*	SMNS 63744	29500	4100	0.14	NA	Southern Germany plattenkalks	shallow	Coleiidae
*Palaeopolycheles longipes*	SMNS 63833	22000	3000	0.14	NA	Southern Germany plattenkalks	shallow	Coleiidae
*Palaeopolycheles longipes*	SMNS 70203	22000	3300	0.15	NA	Southern Germany plattenkalks	shallow	Coleiidae
*Proeryon giganteus*	UJF-ID 11547	71000	11000	0.15	7744	La-Voulte-sur-Rhône	deep	Coleiidae
*Proeryon giganteus*	UJF-ID 14020	79000	11600	0.15	6209	La-Voulte-sur-Rhône	deep	Coleiidae
*Proeryon giganteus*	FSL 170603	85000	9100	0.11	8100	La-Voulte-sur-Rhône	deep	Coleiidae
*Proeryon hartmanni*	BSPG 1942 I 20	60000	6100	0.10	NA	Holzmaden	intermediate	Coleiidae
*Proeryon hartmanni*	SMNS 64019	89000	7700	0.09	NA	Holzmaden	intermediate	Coleiidae
*Rosenfeldia oppeli*	SMNS 66004	38000	3400	0.09	1824	Southern Germany plattenkalks	shallow	unknown
*Soleryon amicalis*	MHNL 20271902	72500	2500	0.03	NA	Cerin	shallow	Eryonidae
*Tethyseryon campanicus*	CSMNF 22000a	21500	2400	0.11	NA	Monte Fallano	shallow	Coleiidae
*Tethyseryon campanicus*	CSMNF 22000c	8200	1100	0.13	NA	Monte Fallano	shallow	Coleiidae
*Tethyseryon campanicus*	CSMNF 22000 g	10000	1200	0.12	NA	Monte Fallano	shallow	Coleiidae
*Willemoesiocaris ovalis*	MNHN F A29521	45000	7000	0.16	4738	La-Voulte-sur-Rhône	deep	Coleiidae
*Willemoesiocaris ovalis*	FSL 170602	32000	5800	0.18	NA	La-Voulte-sur-Rhône	deep	Coleiidae

Distances in μm, surfaces in μm^2^

### Documentation

Small specimens were documented utilizing composite auto-fluorescence imaging [57–59], macro-fluorescence [58, 60] under polarized light (analyzed; [9, 10, 61–63]) by a single-lens reflex camera or a flatbed scanner. Composite images were processed in CombineZM/ZP, Zerene stacker, Microsoft Image Composite Editor or Photoshop CS3. For some specimens detailed close-ups were documented with SEM (Jeol Neoscope 2 JCM 6000). Details of (isopod and insects) comparative material was documented on a Leica stereomicroscope or Zeiss Axioskop 1 equipped with a Skopetek DCM 510 digital camera producing image stacks. Based on these image stacks, virtual surface reconstructions were calculated and presented as red-blue stereo anaglyphs according to the method recently described by Haug et al. [64].

### Calculation of eye parameters

Three values were measured on the high-resolution images:

1. Eye diameter d; due to the often incomplete preservation and possible deformation of the eye, we assumed a roughly circular diameter. We measured the highest distance possible (axis) between two opposing margins for each eyes. In the case of a more oval-shaped outline an average of the two axes was used. In the case of incompletely preserved eyes, the greatest measurable length on the eye was used (Tables 1 and 3).
2. Either 2a) the edge length of a square-shaped facet a or 2b) the diameter of a hexagonal facet a, which equals 2× the edge length (Tables 1, 2 and 3). Since ommatidia edge length is variable across eye surface, we measured the edge length of as many adjacent ommatidia as possible and divided this value by the number of ommatidia. Measurements were made along different axes of the eye and at different places; for all specimens but three (Table 2), variations were below the precision of our measurement or ommatidia were of similar sizes across the preserved portion of eye. The three specimens, in which variations in ommatidia edge length were detected, are detailed in Table 2. For these specimens, mean value of ommatidia edge length was compared with other specimens. We note that the preservation of ommatidia varies. Some ommatidia appear convex, others concave or surrounded by walls. These variations, which may be linked to different preservation, may affect slightly the measurements of ommatidia. This probable bias is unfortunately unavoidable when dealing with fossil material, but considering the excellent preservation of measured ommatidia, this bias is probably limited.
3. The carapace total length, measured from the most anterior to the most posterior point of the carapace, parallel to the longitudinal axis (Table 3).

The exact curvature of the eye surface cannot be measured, as all fossils showed a more or less strong (although sometimes only a slight) compaction. To be as cautious as possible, we considered both possible extreme shapes of an eye which may be either flat or display a hemispherical outline. The original morphology most likely ranged somewhere between these two extremes. In consequence, all further calculations have to be considered estimations, and to refer only to the visible part of the preserved eye, which is the eye most likely formed more than a half sphere, i.e. almost a full sphere; however we have no information on parts below the visible preserved parts.

We then calculated how many ommatidia (n) are present along the longest line across the surface by dividing diameter (assuming a flat eye) or half circumference (πd/2 assuming a hemispherical eye) divided by (a). Based on this, the average opening angle for each ommatidium was calculated as 180° divided by n (for half sphere; opening angle for flat eyes is 0). The surface of the eye was calculated as πd^2^/4 (flat) or πd^2^/2 (hemispherical). This value was divided by the surface of a single ommatidium (a^2^, for square-shaped; ((3√3)/2)(a/2)^2^, for hexagonal) to get the total number of ommatidia per visible surface of the eye.

### Statistics

To study the possible impact of palaeoenvironments and phylogenetic on eyes parameters, we performed a series of statistical analysis using R (http://www.R-project.org). Measured values were first tested for normality (Shapiro test) for whole samples (eye size, ommatidia) and in subset defined by categories (familial assignment, outcrops); not all subsets were normally distributed, for this reason, non-parametric tests (considered more robust for not normally distributed samples) were used for analysis of the whole sample and a parametric test two compare normally distributed subsets (Coleiidae and Eryonidae ratios—see below; parametric tests are considered more valid when applied to normally distributed samples). We compared the diameter of eyes to the carapace length to search possible link between the two variables (Spearman correlation). Subsequent test were based on the ratio “eye diameter” on “carapace length” and the possible influence of palaeoenvironments (outcrops) and phylogeny (familial assignment) (Kruskal-Wallis tests). Ratios of “eye diameter” to “carapace length” were compared between Eryonidae and Coleiidae, since number of specimens for other families is not sufficient for statistical analysis. Size of ommatidia was also compared to the diameter of the eyes. We used the square root of ommatidia surface to normalize the size of ommatidia and compare its variation to that of the diameter of the eye (spearman correlation).

### Phylogenetic context

Our evolutionary scenario is based on the phylogenies of Scholtz & Richter [31] and Ahyong [43]. Familial assignments follow Audo et al. [24], and Ahyong [43], except in the case of *Rosenfeldia oppeli*, which due to its unusual appearance is not considered as an eryonid.

## Results

### Eyes and ommatidia

All specimens examined possess anterior pairs of ball- or disk-shaped structures that strongly resemble stalked compound eyes of extant decapod taxa. On 14 specimens, these protuberances display either squared or hexagonal sculpturations. On the remaining specimens, the preservation is not as fine, and these fine sculpturations are not visible. The nature of sculpturations is slightly obscured by compression in flattened specimens. However, objects with fragmentary volume preservation, as for instance in specimens from La Voulte, can be confidently interpreted as fossilized compound eyes. Due to the specific preservation of these eyes, some being partly flattened, incomplete and/or not visible in their entirety, detailed descriptions are difficult. Ommatidia do usually not cover the entire surface. Therefore, the descriptions are used as a basis for simple geometric assumptions and generalizations about the entire eye. Measurements and estimations of the properties of these eye structures are summarized in Table 1, see also Table 2.

We find quite a variety of ommatidia sizes from 35–90 μm, their inferred number also varies considerably; depending on high or low estimation there are about 700–17.000 to 1500–34.000 ommatidia per eye.

### Differences in eye sizes

**Fig. 9:**
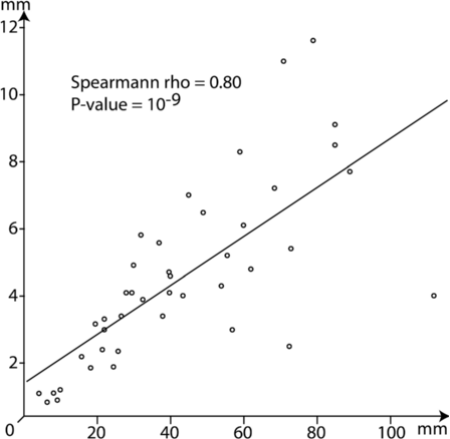
Scatterplot of the diameter of the eye and the length of carapace with regression line

Comparison between the diameter of the eye and carapace length shows that both values are linearly linked (Spearmann rho = 0.80; *P*-value < 10^−9^: Fig. 9). This suggests, as expected, that larger specimens have larger eyes.

**Fig. 10:**
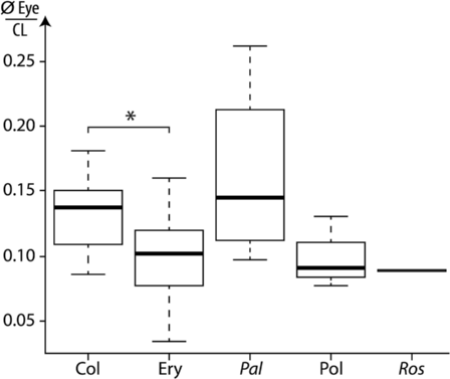
Boxplot comparing the ratio of the eye diameter on the carapace length (CL) in five different groups of fossil polychelidans: Col = Coleiidae, Ery = Eryonidae, *Pal* = *Palaeopentacheles* (only species of Palaeopentachelidae), Pol = fossil species closely related to Polychelidae, *Ros* = *Rosenfeldia oppeli*. *, Welch test, *P*-value < 0,01. Abbreviations

Difference in the proportion of the eyes (diameter of the eye = Eye Ø), relatively to the carapace length (CL) cannot be explained by variations in palaeoenvironments based on our data (Kruskal-Wallis χ^2^ = 6.95; *P* = 0.43). However, phylogenetic variations may help to explain differences in eyes proportion (Kruskal-Wallis χ^2^ = 11.61; *P* < 0.05). More precisely, representation of the distribution of values for different families (Fig. 10) shows that: Coleiidae have in proportion larger eyes than Eryonidae and that fossil species more closely related to the modern species of Polychelidae than to fossil species attributed to other families;

- Palaeopentachelidae have in proportion larger eyes than all other taxa (Note that this family is currently composed of a single species: *Palaeopentacheles roettenbacheri*);
- Eryonidae, stem-Polychelidae and *Rosenfeldia oppeli* have eyes of similar proportions.

We compared statistically Eryonidae to Coleiidae (sample-size for other subgroups – here families – is insufficient). Eryonidae eyes are significantly smaller than those of Coleiidae (Welch test: Df = 30.8; *P* < 0.01).

### Ommatidial size

**Fig. 11:**
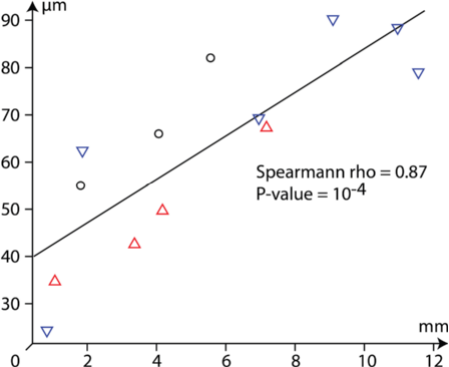
Scatterplot of the square root of ommatidial surface and the diameter of the eye: blue triangles, specimens from La Voulte (probably deep); red triangles, specimens from plattenkalks (probably shallow); circles, specimens from other outcrops

Comparison between the square root of ommatidial surface and diameter of the eye shows that both values are linearly linked (Spearmann rho = 0.87; *P* < 10^−4^: Fig. 11). This implies that large eyes include larger ommatidia. Ommatidia of La Voulte species (Fig. 11) are generally slightly larger than other, unfortunately, our sample-size doesn’t allow for detailed comparisons between families or outcrops, so these differences cannot be distinguished from random variations. The small sample size and, as indicated above, variations in the preservation of ommatidia may also have obscured the palaeoenvironmental signal.

## Discussion

### Optical mechanisms of crustacean eyes and interpretation of fossil polychelidan eyes

Generally, arthropod compound eyes have been classified to function according to two common optical principles: the ancestral apposition type and superposition type which most likely evolved several times in parallel. In apposition eyes, every separate element of the compound eye (ommatidium) acts as independent optical unit. In the different types of superposition the cornea of each ommatidium may spread light onto photoreceptor cells of adjacent ommatidia, thus forming a superimposed image. Apposition eyes are optimized for maximal optical resolution whereas superposition optics increase absolute sensitivity of the eyes [65]. In modern crustaceans, and specifically in decapods, a high diversity of different superposition eye types is found in adults: refracting, reflecting, and parabolic superposition optics [65–70]. Although being quite diverse in adults, larval decapod compound eyes are exclusively of the apposition type [65, 71–74]. The evolutionary transitions of these optical types within Decapoda in general and in Reptantia in particular have been addressed by Gaten [69] and Richter [70]. Gaten [69] did not discuss Polychelida. Richter [70] considered the poylchelid stem species to be blind based on representatives of extant polychelids only. According to our analysis of fossil representatives with preserved eye structures, the view proposed by Richter [70] needs to be revisited.

### Interpretation of structures: square ommatidia

We found spherical or subspherical protuberances covered by a distinctive sculpturation of squares or hexagons at the anterior end of the cephalothorax of various Mesozoic polychelids that we interpret as fossilized compound eyes. This interpretation is based on the position, shape and specific surface of these structures in comparison to the compound eyes of extant euarthropods. In all examined species (except *Rosenfeldia oppeli*), compound eyes with square facets are present. This morphology is suggestive of reflective superposition [65], an optical mechanism in which the light received by a given ommatidium is reflected through a “mirror box” onto the light-guiding structures of neighbouring ommatidia as well. Indeed, the square shape of the ommatidial prism allows two reflections of light to enter the ommatidia against prism walls, and giving rise to an erected image, which allows superposition without changes in the refractive index. Extant representatives of the Palinuridea (also assigned to reptantian decapods) do also possess square facets and reflexive superposition, e.g. representatives of the genera *Jasus* [75] and *Panulirus* [76]. An alternative for this interpretation, although unlikely exists: indeed, some xanthid crabs (e.g. *Trapezia* spp.) also possess square ommatidial facets, but their eyes are of the parabolic superposition type [65, 67]. Because neither the longitudinal profile of the crystalline cone nor its refractive gradients are preserved during fossilization, we cannot entirely exclude the possibility that early polychelidans had parabolic superposition eyes, in analogy to these examples.

The finding of reflective superposition eyes in different representatives of Polychelida closes the gap of knowledge in the early evolution of reptantian decapods [70]. Adult reflecting superposition eyes appear to have been retained from the ground pattern of Decapoda along the entire early evolutionary lineage of Polychelida. Reduced compound eyes are so far known only known in one subgroup: Polychelidae. Therefore, blindness, as found in extant Polychelidae, becomes even more evidently a result of secondary evolution.

### Eye preservation

The described specimens here mark the oldest direct evidence of superposition eyes within euarthropods. The presence of reflective superposition eyes is remarkable from the aspect of preservation of eye structures.

In many cases in which compound eyes are fossilized, the ommatidia are either recognizable as 1) dome-shaped swellings on the surface or 2) cavities. In these two cases, apparently inner structures of the ommatidium play a major role in the preservation. In the former case, the swelling may represent an original structural aspect. If we compare it to, for example, SEM images of many extant arthropod compound eyes, we see that similar dome-shaped swellings mark the ommatidia. Alternatively, the ommatidium can be partly collapsed (due to taphonomic processes) but become stabilized by inner structures of the ommatidium. If preserved as a cavity, the outer “walls” of the ommatidium must preserve its stability.

In specimens presented here, such a type of preservation appears to be found in isopods and the insect specimens (Figs. 7 and 8). Among the polychelidan specimens, the single specimen of *Palaeopentacheles roettenbacheri* may possess this type of preservation. Some of the more roughly preserved eyes of some of the polychelidans (Figs. 3a-d; 6a-d) may represent taphonomically collapsed ommatidia. In some specimens from La Voulte, the cornea was probably broken during preparation of the fossil; hence it is likely that what we have observed here is the inner part of the inner lumen of the ommatidia.

In most of the other specimens, the preservation appears to be different and we most likely see the fossilized cornea. In extant decapod crustaceans (e.g. Astacida, Caridea, Galatheoidea, some Brachyura), the square-shaped cornea does reflect the square cross-shape of the crystalline cone in the ommatidia [69, 75, 77–79]). Occasionally, as for instance in some dendrobranchiate crustaceans (prawns), corneal facets may vary considerably in shape, ranging diffusely between square, pentagonal and hexagonal facets, especially to the margins of the eye [80]. This is caused by simple geometrical constraints on a curved surface. The structural variability of the surface is continued further proximally in the ommatidium, hence the crystalline cones display variable cross-profiles. Despite their structural variability, compound eyes of Dendrobranchiata have been interpreted as representing reflective superposition eyes [69, 70]. Strong variations also occur in compound eyes with hexagonal facets, such as the refractive superposition eyes of some mysidacean crustaceans (compare with Richter [80]). This indicates that even the frequent correlation of square facets and reflective superposition may be obscured, at least in some malacostracan taxa.

### Hexagonal ommatidia of Rosenfeldia oppeli

In *Rosenfeldia oppeli*, with its hexagonal facets, it is even more difficult to judge from external characters alone which optical type this eye represents. Hexagonal facets occur in different types of eyes with different optical mechanisms. Indeed, eyes with hexagonal facets are known to occur [65, 67, 81]:

1. In apposition eyes, including larval apposition eyes, which may eventually be replaced by superposition eyes later in development in some decapod taxa;
2. In refractive superposition eyes;
3. In parabolic superposition eyes.

Our careful examination of the specimen of *Rosenfeldia oppeli* only allowed the description of the external aspect. Therefore, it is not possible to distinguish with confidence to which optical type *R. oppeli* hexagonal ommatidia correspond. However, it is possible to provide some background on the probability of each of these possibilities:

1. Apposition optics in adult compound eyes are part of the ground pattern of Malacostraca [70]. Thus, their occurrence is not unexpected in any malacostracan crustacean. However, within Eumalacostraca, refractive or reflective superposition eyes independently evolved several times [70] and therefore cannot be excluded here for *R. oppeli* (see next points). The multiple evolution of different types of superposition in adult crustaceans is linked to alterations of ommatidia adjusted to perform apposition as larvae. However, Nilsson et al. [82] showed that for instance the transformation of apposition into refracting superposition does not require drastic changes of the ommatidial set-up, neither on the cellular nor on the subcellular level. Therefore, the optic apparatus of decapods is likewise prone to convergent modifications of inherited structures, even though these incidents, such as the evolution of refracting superposition, apparently happened less often than theoretically possible [70]. Alternatively, the specimen could represent a larva with apposition eyes because this type is found in all extant malacostracan larvae [65, 71–74]. However, the specimen of *R. oppeli* studied here seems too large (carapace length = 38 mm) to be a larva. Therefore, the occurrence of apposition eyes in this specimen would be best explained by paedomorphosis.
2. Refractive superposition eyes are frequent within Malacostraca [70]. Although the ground pattern of Decapoda is characterized by reflective superposition eyes, several ingroups, such as representatives of the shrimp genus *Gennadas* possess well-functioning refracting superposition eyes with hexagonal facets. The compound eyes of *R. oppeli* may thus represent this type of eye. However, among decapod crustaceans, reflecting superposition, as characterized by square facets and mirror boxes [65, 83, 84], are frequently encountered. Depending on the phylogenetic concept applied, reflecting superposition optics either evolved in the stem lineage of or very early within Decapoda [70]. Variations of superposition optics, namely the occurrence of refracting superposition in a context of species with reflecting superposition eyes, are not very common in a given decapod taxon but they do occur occasionally. Examples are representatives of the shrimp genus *Gennadas* which possess well-functioning refracting superposition eyes with hexagonal facets, but their closest ancestors had reflecting superposition eyes [68, 85].
3. Parabolic superposition eyes occur in a few groups of decapod crustaceans, such as Xanthidae and Portunidae (e.g. *Macropipus depurator*) [65, 67, 85]. However, if present, their distribution among decapod crustaceans would most likely imply a convergent evolution of such eyes for polychelidan lobsters.

By comparison with the eyes described for other polychelidan lobsters described in the present study, it seems more plausible that the eyes of *R. oppeli* correspond either to apposition eyes derived from the transparent subtype and retained though paedomorphosis or they represent refractive superposition eyes. The occurrence of possibly paedomorphic apposition eyes or refractive superposition eyes of *Rosenfeldia oppeli* is most likely an autapomorphy of this species.

### Visual palaeoecology

Eyes of crustaceans possess a wide array of adaptations to different environments and light intensities: the size of ommatidia can vary; superposition eyes allow sensitivity to light while apposition eyes have a higher resolution; in all cases, pigments can modulate the light entering the ommatidia or isolate ommatidia one from another in superposition eyes; reflective layers (proximal tapetum) can also enhance sensitivity by reflecting the light in the eye so it passes twice through the retina [85, 86].

From the fossil perspective, most of the adaptations of eyes are hidden in external view and possibly not preserved in fossil for which only the superficial lenses are available (for instance, in exuviae). However, the shape and size of ommatidia lenses are readily observable.

In the case of *R. oppeli*, if we consider its hexagonal facets to be indicative of apposition eyes, we can draw some conclusions regarding its life-habits: indeed, apposition eyes are by far less sensitive to light than superposition eyes, because the light illuminating the cornea can only come from one facet, and is correlated to its surface. However, these eyes have a higher resolution than that of superposition eyes, because each ommatidium is independent.

In the case of the other species described here, interpretation of their life-habits from their eyes is more complex. All of them possess reflecting superposition eyes and differences between those eyes can only apprehended from our perspective through the size of ommatidia and the size of the eye. Our observations show that the size of eyes is correlated to the size of the carapace (and thus to that of the animal). Differences in visual performance between bright-light-adapted and dim-light-adapted species could be expected to be observed from the proportion of the eye compared to the carapace. However, our analysis did not reveal any significant differences in this ratio between the various groups. Significant differences are however observed between different taxa of polychelidan lobsters.

Multiple factors may explain this apparent lack of correlation:

- Our sample of preserved ommatidia is too small, or variations in the preservation, add noise to a possible palaeobathymetric signal.
- Differences in palaeobathymetry of the various outcrops we sampled may not be sufficient to observe differences in the size of eyes.
- Other mechanisms are probably involved (such as the presence of pigments reducing light input in shallow water species and tapetum reflecting the light through the retina in deep-water species).
- Most extant shallow-water crustaceans tend to hide during the day and search for food at night; for this reason, they may need ommatidia as large as deep water species.

## Conclusions

- Reflective superposition eyes can be preserved in fossil arthropods.
- Modern representatives of Polychelida are blind; Jurassic representatives still possessed functional eyes, most of them reflective superposition eyes retained from the ground pattern of Decapoda.
- The examples presented here mark the oldest direct evidence for reflective superposition eyes and of clear superposition eyes in general.
- The eye of *R. oppeli* could be an apposition eye that evolved through paedomorphic heterochrony. *R. oppeli* might have lived in shallower water than its relatives. Alternatively, it could correspond to a refractive or even a parabolic superposition eye.
- Eyes proportions appear linked to phylogeny.

We hope that with the reinvestigation of other fossils we may complete these observations and obtain a clearer picture of how variations in eye design and size is distributed across taxa of polychelidans and if a variation between outcrops can be observed.

## Abbreviations

CSMNF, Centro Musei delle Scienze Naturali e Fisiche of the Università degli Studi di Napoli “Federico II” (Napoli, Italy); FSL, Université Claude Bernard Lyon 1 (Villeurbanne, France); JME-SOS, Jura-Museum Eichstätt (Eichstätt, Germany); MNHN.F, Muséum national d’Histoire naturelle, collection de Paléontologie (Paris, France); MSNM, Museo Civico di Storia Naturale di Milano (Milano, Italy); NHMUK, Natural History Museum (London, United Kingdom); SMCU, Sedgwick Museum – Cambridge University (Cambridge, United Kingdom); SMNS, Staatliches Museum für Naturkunde (Stuttgart, Germany); SNSB-BSPG, Bayerische Staatssammlung für Paläontologie und Geologie (Munich, Germany); UJF-ID, Université Joseph Fourier, Institut Dolomieu (Grenoble, France); UPMC, Université Pierre et Marie Curie – Paris 6 (Paris, France)
